# Mitochondria as the Linchpin of General Intelligence and the Link between *g*, Health, and Aging

**DOI:** 10.3390/jintelligence7040025

**Published:** 2019-11-25

**Authors:** David C. Geary

**Affiliations:** Department of Psychological Sciences, Interdisciplinary Neuroscience, University of Missouri, Columbia, MO 65211-2500, USA; GearyD@Missouri.edu; Tel.: +573-882-6268; Fax: +573-882-7710

**Keywords:** general intelligence, mitochondria, cognitive aging, health

## Abstract

In a recent theoretical article, I proposed that the efficiency of mitochondrial functioning is the most fundamental biological mechanism contributing to individual differences in general intelligence (*g*; Geary, 2018). The hypothesis accommodates other contributing mechanisms at higher levels of analysis (e.g., brain networks), and is attractive because mitochondrial energy production undergirds the developmental, maintenance, and expression of these other mechanisms and provides a means to link individual differences in *g* to individual differences in health and successful aging in adulthood. I provide a brief summation here and a few clarifications to the original article.

It has been well over a century since Spearman discovered that individuals who perform well in one cognitive or academic domain generally perform well in all other domains, leading him to conclude "that all branches of intellectual activity have in common one fundamental function (or group of functions)" [1] (p. 285), which he termed general intelligence, or *g*. Statistically, about 50% of the covariance among cognitive and academic measures is captured by this function or functions [2], making the concept of *g* (whatever it is indexing) of substantial theoretical and practical importance. The ensuing search for the basis of *g* has led to myriad theories and research traditions at multiple levels of analysis including complex (e.g., working memory) to more basic (e.g., speed of processing) cognitive systems [3,4]; complex (large-scale networks) to more basic (e.g., speed of neural conduction) neural systems [5]; aspects of cellular functioning (e.g., control of oxidative stress; [6,7]); and, g as an illusionary construct resulting from the mathematics underlying factor analyses [8,9]. 

In a recent proposal, I suggested that *g* is best conceptualized as a group of cognitive and neural functions that are all undergirded by one evolutionarily old, fundamental function [10]; specifically, the efficiency of various mitochondrial processes, especially the production of cellular energy. The group of functions means that multiple cognitive and brain systems contribute to *g* in a nested fashion, as shown in Figure 1. The outer level is represented by the engagement of the cognitive systems, such as working memory, and problem-solving approaches, such as analogical reasoning (e.g., [11]), that manifest in the real world as intelligent decision making and behavior. It is now well established that these competencies are supported by distributed and dynamically interacting networks of brain systems (the intermodular ring in Figure 1), although the engaged networks likely depend on task demands and the individuals’ level of expertise in the area ([5,12,13]). The efficiency of these complex intermodular systems will necessarily be modulated by the efficiency of the intramodular systems that compose them [14], and these in turn are dependent on the functioning of the constituent neurons and supporting cells (glia; [15]). At the core are mitochondria and their many functions, including the bulk of cellular energy production [16]. Genetic, neuroimaging, and neuropsychological studies can be marshalled to support each of these mechanisms as contributing to *g* [17,18,19], but none of them in and of itself will likely provide a full explanation.

A core implication of the nested structure shown in Figure 1 is that deficits or inefficiencies at lower levels will ripple through all higher levels but deficits at higher levels (e.g., resulting from traumatic brain injury) need not have broad influences at lower levels. In other words, the full expression of the competencies supported at higher levels, within the genetic and experiential constraints of the individual, will be limited by the efficiency of systems at all lower levels. On this view, subtle variation—either due to genetics [20] or experiences (e.g., toxin exposure, chronic stress; [21])—in mitochondrial energy production or the mechanisms that protect mitochondria from degradation will manifest as variation in the development and expression of all of the brain and cognitive systems that have been linked to *g*. 

Critically, mitochondria produce the majority of energy consumed by all biological systems, not just the brain, and the initial tranche of mitochondria within all of these systems comes from the same limited pool. As a result, variation in the efficiency (e.g., in energy production, control of oxidative stress) of the initial pool of mitochondria will be expressed throughout the body and will contribute to individual differences in resilience to disease and stressors—factors that can degrade mitochondrial functioning—and can influence the rate of aging in adulthood [22,23,24]. Variation in the initial pool of mitochondria also provides a straightforward explanation of the well-documented relations among *g*, general health, and successful aging in adulthood [25,26,27]. 

As just one example of these relations, there is a single statistical factor that explains individual differences in rate of age-related declines across cognitive domains (e.g., reasoning, speed of processing; [28]). As people age, individuals who show sharp performance declines in one area, such as fluid intelligence, also show parallel declines in other cognitive domains, such as speed of processing. The implication is there is a single mechanism (or group of related mechanisms) that supports cognition and declines as a natural consequence of biological aging. The statistical factor that captures this common age-related decline is moderately correlated (*r* ~0.5) with an estimate of *g* [29]. In other words, there is overlap in the mechanisms that contribute to *g* and to natural age-related declines in cognition. These types of studies, however, are not evidence that the overlap is the result of a single mechanism, as this is unlikely. 

Normal age-related declines in mitochondrial energy production is a very plausible contributing mechanism, even if it is not the whole story. Age-related decrements in energy production will occur, at least to some extent, in parallel across biological systems and individual differences in the rate of change will result in correlations across cognitive and health measures. Moreover, the relative importance of mitochondrial functions in relation to other factors might increase with aging in adulthood. The increasing importance of mitochondria follows from its role as a limiting mechanism on the functioning of more complex systems. With normal age-related declines in energy production, an increasing number of individuals will have energy-production levels that approach or drop below the thresholds needed to maintain and express higher-level systems at their optimal; that is, optimal for the individual depending on the genetic and experiential factors that contribute to the construction of these systems, given sufficient energy levels. 

Stated somewhat differently, the capacity for mitochondria to produce energy places a ceiling on the performance of higher-level systems. With normal aging in adulthood that ceiling slowly descends and reduces the capacity for higher-level systems to operate at levels they once did. These declines would be related to reductions in the ability to maintain the complex brain systems that support intelligence and in the ability to use them as effectively during periods of high-energy demand, such as maintaining focus during a novel and complex problem-solving task. During development individual differences in mitochondrial energy production are likely to place constraints on the construction of these systems in addition to constraints on optimal functioning. In this latter case, the overall ceiling is higher and thus variation in *g* might be more strongly related to variation in higher-level than lower-level systems, but this remains to be determined.

## Figures and Tables

**Figure 1:**
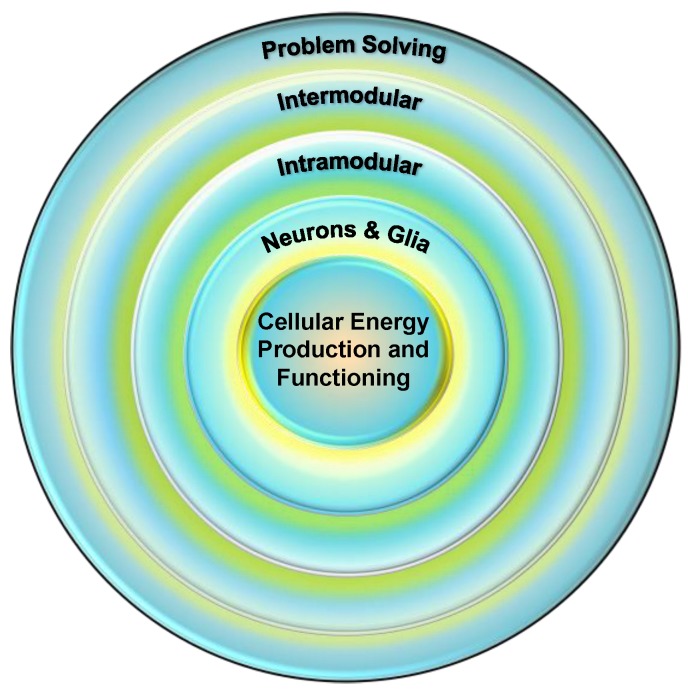
Individual differences in *g* are likely to be influenced by the functioning of multiple cognitive and brain systems, the optimal functioning of which is dependent on systems below it. Cellular energy is the lowest common currency driving the development and expression of all biological systems and thus places upper-limit constraints on the development and expression of all other systems.
